# Dr. Lemier Congdon

**Published:** 1909-12

**Authors:** 


Dr. Lemier Congdon, of Springfield, Mass., died at his home in that city, November 4. 1909, aged 74 years. He graduated at the Medical Department, University of Buffalo, in 1852.

